# Insights into the architecture of human-induced polygenic selection in Duroc pigs

**DOI:** 10.1186/s40104-022-00751-x

**Published:** 2022-09-21

**Authors:** Zitao Chen, Jinyan Teng, Shuqi Diao, Zhiting Xu, Shaopan Ye, Dingjie Qiu, Zhe Zhang, Yuchun Pan, Jiaqi Li, Qin Zhang, Zhe Zhang

**Affiliations:** 1 National Engineering Research Center for Breeding Swine Industry, Guangdong Provincial Key Lab of Agro-Animal Genomics and Molecular Breeding, College of Animal Science, South China Agricultural University, Guangzhou, 510642 P.R. China; 2 Department of Animal Science, College of Animal Science, Zhejiang University, 866# Yuhangtang Road, Hangzhou, 310058 P.R. China; 3 Guangdong Provincial Key Laboratory of Marine Biotechnology, Shantou University, 243 Daxue Road, Shantou, 515063 P.R. China; 4 Fujian Yongcheng Agricultural & Animal Husbandry Sci-Tech Group Co., Ltd., Fuqing, 350399 P.R. China; 5 Department of Animal Genetics and Breeding, College of Animal Science and Technology, Shandong Agricultural University, Tai’an, 271018 P.R. China

**Keywords:** Artificial selection, GWAS, Pig, Reproductive organ, Selection signatures

## Abstract

**Background:**

As one of the most utilized commercial composite boar lines, Duroc pigs have been introduced to China and undergone strongly human-induced selection over the past decades. However, the efficiencies and limitations of previous breeding of Chinese Duroc pigs are largely understudied. The objective of this study was to uncover directional polygenic selection in the Duroc pig genome, and investigate points overlooked in the past breeding process.

**Results:**

Here, we utilized the Generation Proxy Selection Mapping (GPSM) on a dataset of 1067 Duroc pigs with 8,766,074 imputed SNPs. GPSM detected a total of 5649 putative SNPs actively under selection in the Chinese Duroc pig population, and the potential functions of the selection regions were mainly related to production, meat and carcass traits. Meanwhile, we observed that the allele frequency of variants related to teat number (NT) relevant traits was also changed, which might be influenced by genes that had pleiotropic effects. First, we identified the direction of selection on NT traits by $\hat{G}$, and further pinpointed large-effect genomic regions associated with NT relevant traits by selection signature and GWAS. Combining results of NT relevant traits-specific selection signatures and GWAS, we found three common genome regions, which were overlapped with QTLs related to production, meat and carcass traits besides “teat number” QTLs. This implied that there were some pleiotropic variants underlying NT and economic traits. We further found that rs346331089 has pleiotropic effects on NT and economic traits, e.g., litter size at weaning (LSW), litter weight at weaning (LWW), days to 100 kg (D100), backfat thickness at 100 kg (B100), and loin muscle area at 100 kg (L100) traits.

**Conclusions:**

The selected loci that we identified across methods displayed the past breeding process of Chinese Duroc pigs, and our findings could be used to inform future breeding decision.

**Supplementary Information:**

The online version contains supplementary material available at 10.1186/s40104-022-00751-x.

## Introduction

About 10,000 years ago, pigs were domesticated in multiple locations around the world [1]. And then, high-intensity artificial selection has been applied to the genetic improvement of agriculturally important traits [2]. As selection at favorable mutations have played an essential role in the domestication and genetic improvement of animals, the frequency of favorable mutations will increase rapidly and this process is called selective sweep. Different approaches have been proposed for the identification of selective sweep, e.g., the genetic diversity ratio (θ_π_) and Wright’s fixation index (*F*_ST_) [3]. Most approaches are designed to identify genomic regions with large-effect and have successfully identified large-effect quantitative trait loci (QTL) under selection that controls pig traits, e.g., coat color, meat quality, and fertility [4]. Indeed, agriculturally important traits are usually controlled by many mutations of small effect.

Recently, some methods have been developed to detect polygenic selection, e.g., the Generation Proxy Selection Mapping (GPSM) allows us to observe how complex polygenic selection alters the genome over short timescales in a trait-agnostic manner [5] and $\hat{G}$ can be used to powerfully identify selection on highly polygenic traits [6]. The GPSM method uses a proxy for generation number, e.g., birth date, as the dependent variable in a genome-wide linear mixed model to detect associations between generation and allele frequency caused by ongoing selection. Meanwhile, $\hat{G}$ uses pre- and post-selection genotypic data with a single time point with phenotypic information to identify selection on traits that are controlled by many genes.

Duroc pig, as an older breed of domestic pig, was developed in America and formed after a long period of artificial selection. In modern pig industry, Duroc pig is one of the most utilized commercial composite boar lines, and well-known for its growth, feed conversion efficiency, carcass and meat quality traits [7]. Because pleiotropic function exerts, high-intensity artificial selection on production traits potentially causes the weakening of other traits. For instance, the average teat number of Duroc pig breed was lower than that of the Large White [8] and Landrace pig breeds [9].

Herein, we first used two methods (GPSM and $\hat{G}$) to detect ongoing polygenic selection in a factory-farmed Duroc pigs. Further, runs of homozygosity (ROH) was complementally detected to explore the selection landscape. Then, we performed genome-wide association studies (GWAS) and selection-mapping protocols (*F*_ST_ and θ_π_ ratio) to identify the potential large-effect NT trait-related genomic regions. Further, we conducted a comprehensive analysis to identify the putative pleiotropic genomic regions. The results of this study uncover the genetic improvement of Chinese Duroc pig population over the past decade and will be used to inform future breeding decision.

## Materials and methods

### Ethical statement

All experiments in this study were approved by the Animal Care Committee of South China Agricultural University (Guangzhou, People’s Republic of China) with approval number SCAU#2013-10, and the experiments were performed according to the regulations and guidelines established by this committee.

### Sample preparation and sequencing

A total of 1067 animals consisted of 984 females and 83 uncastrated males from a Duroc pig population managed in Fujian, China, were used in this study. These animals were born between 2009 and 2017. All phenotypic records were extracted from the Herdsman swine management platform (S&S Programming, Lafayette, IN, USA). The number of left teats, right teats were recorded by simple counting. The number of teats was the sum of the teat number at both sides. In addition, the number of left teats, right teats, and teats of each individual was counted at birth and the malformed teats were not recorded. Furthermore, we obtained the phenotypic (litter size at weaning (LSW), litter weight at weaning (LWW), days to 100 kg (D100), backfat thickness at 100 kg (B100), and loin muscle area at 100 kg (L100)) data from our previous studies [10, 11].

In this study, we extracted genomic DNA from the ear tissue of 1067 Duroc pigs using the TaKaRa MiniBEST Universal Genomic DNA Extraction Kit (Version 4.0), then checked using agarose gel electrophoresis and quantified with a NanoDrop 2000 (Thermo Scientific, Waltham, MA, USA). Either the Illumina PorcineSNP60 BeadChip (Illumina, San Diego, CA, USA) comprising 63,480 SNPs or the GeneSeek GGP-Porcine chip (Neogen Corporation, Lansing, MI, USA) comprising 51,558 SNPs were used to genotype the individuals. The common SNPs contained 33,359 SNPs between two chips were retained. Among the Duroc pigs used in the current study, we selected 50 key individuals using the marker-based genetic relationship matrix to maximize the expected genetic relationship between the key individuals and the remaining population, as in Ye et al. [12]. These individuals were re-sequenced with 150 bp paired-end reads using the Illumina HiSeq 3000 platform. In the raw reads, the adaptor polluted reads and multiple N reads (where *N* > 10% of one read) were removed using Trim Galore version 0.6.1 to produce the clean reads. Further, the clean data were aligned to the *Sus scrofa* 11.1 reference genome using Burrows-Wheeler Aligner (BWA) version 0.7.15 [13]. The genome analysis toolkit GATK version 4.1.2.0 [14] was used to detect the SNPs using a Bayesian model, a total of 19,754,293 SNPs was found. Subsequently, genotype imputation was performed, treating 50 key individuals with sequencing data as reference panel, and the remaining 1017 individuals with SNP arrays data were imputed to whole genome sequencing (WGS) data using Beagle version 5.0 [15]. Quality control of SNPs was implemented using VCFtools version 0.1.14 [16], following the below criteria: retaining (1) the SNPs with a DR^2^ ≥ 0.8; (2) the SNPs with a call rate ≥ 0.9 or MAF ≥ 0.01 or significant deviations from Hardy-Weinberg equilibrium (*P*-value ≥ 0.00001); (3) retaining sites with a mean depth < 3. Hence, a total of 1067 individuals with 8,766,074 independent SNPs were eligible for inclusion in the following analyses (Fig. S1). The genotype concordance rate, defined as the proportion of identical genotypes between the imputed variants and the whole-genome sequence variants, was 0.96 ± 0.13 across the autosomes. PLINK version 1.09 [17] was utilized to convert file formats of the independent SNPs from variant call format (VCF) to PLINK binary format.

### Detecting of polygenic selection

An animal’s age as of December 21, 2017 was used as the generation proxy in GPSM, and we fit a univariate genome-wide linear mixed model as follows:

$$\boldsymbol Y\boldsymbol=\boldsymbol X\boldsymbol\beta\boldsymbol+\boldsymbol Z\boldsymbol u\boldsymbol+\boldsymbol e$$

Where ***Y*** is an individual’s generation proxy; ***β*** is the estimated effect size for each SNPs; ***u*** is polygenic term and is set as $\boldsymbol u\sim\mathbf N\left(\mathbf0,\boldsymbol G\boldsymbol\sigma_{\boldsymbol a}^{\mathbf2}\right)$, where ***G*** is the genomic relationship matrix. ***X*** and ***Z*** are incidence matrices for ***β*** and ***u***; ***e*** is the random residuals and is set as $\boldsymbol e\sim\mathbf N\left(\mathbf0,\boldsymbol I\boldsymbol\sigma_{\boldsymbol e}^{\mathbf2}\right)$, where ***I*** is an identity matrix. We used FDR corrected *q*-values to control for multiple-testing and SNPs with *q*-value < 0.1 was deemed to be significant variants [5].

We estimated a composite statistic $\hat{G}$ on left teats, right teats, and total teats traits to test for the direction of selection of NT relevant traits. $\hat{G}$ was generated from the relationship between additive effect estimates and allele frequency changes over time. We fit a ridge regression best linear unbiased prediction (RRBLUP) model with NT traits as the response. In RRBLUP model, the fixed effects included year-season of individuals at birth, in which the measured seasons contained four levels (1st = December to February; 2nd = March to May; 3rd = June to August; 4th = September to November). We extracted its estimated effect from the RRBLUP model and changing in allele frequency from 2009 to 2017. Then, $\hat{G}$ was calculated as follows:

$$\hat{\boldsymbol{G}}={\sum}_{\boldsymbol{j}=\mathbf{1}}^{\boldsymbol{m}}\boldsymbol{\Delta} \boldsymbol{j}\boldsymbol{\alpha } \boldsymbol{j}$$

where **Δ*j*** is the change in allele frequency of SNP *j* from 2009 to 2017, ***αj*** is the allele effect of SNP *j*, and *m* is the total number of SNPs. Later, we permuted SNP allele effects with 1000 times to generate $\hat{G} perm$, and compared $\hat{G}$ with $\hat{G} perm$ to test whether the observed composite statistic was the result of selection rather than drift.

### Selection-mapping protocols

We conducted ROH detection to identify regions of homozygosity using PLINK version 1.09 [17] by a sliding window method with the following parameters: (1) a sliding window of 50 SNPs and one heterozygous genotype was allowed in a window; (2) the minimum length for an ROH was set to 1 Mb; (3) the required minimum SNP density was set as 1 SNP per 50 kb; and (4) each ROH contained at least 65 consecutive SNPs. The percentage of SNP occurrences in ROHs was calculated to characterize the genomic regions of ROH hotspots, and the threshold of ROH hotspots was set as the top 0.5% of the SNP occurrences.

The pairwise difference between the individuals born on each year were tested using a Welch Two Sample *t*-test and listed in Table S1. There were significant differences in the number of teats between sows born on 2009–2011 and born after 2011. To further detect NT relevant traits-specific selection signatures, a phenotypic differential population pair, (1) larger teat number (LT) group: 45 sows born before 2011 with larger number of teats (14.16 ± 0.01) and (2) smaller teat number (ST) group: 45 sows born on 2017 with smaller number of teats (12.00 ± 0.00), was created. The θ_π_ ratio and *F*_ST_ were used to detect signatures of selection in this phenotypic differential population pair with the use of a sliding window method (50 kb window and 10 kb step). The θ_π_ ratio between LT group and ST group was calculated as ln(θ_π|LT_/θ_π|ST_). In addition, the 1% of windows with the highest θ_π_ ratio and *F*_ST_ values was considered as the potential selection regions.

### Association analyses

GWAS was performed using a mixed linear model, as follows:

$$\boldsymbol Y\mathit=\boldsymbol W\boldsymbol\alpha\mathit+\boldsymbol X\boldsymbol\beta\mathit+\boldsymbol Z\boldsymbol u\mathit+\boldsymbol e$$

where ***Y*** is the number of left teats, right teats, and total teats of the individuals; ***α*** is a vector of fixed effect, including the year and season of individuals at birth; ***β*** is the substitution effect of the SNPs; ***u*** is the random effect and is set as $\boldsymbol u\sim\mathbf N\left(\mathbf0,\boldsymbol G\boldsymbol\sigma_{\boldsymbol a}^{\mathbf2}\right)$, where ***G*** is the genomic relationship matrix; ***W***, ***X***, and ***Z*** are incidence matrices for ***α***, ***β***, and ***u***; ***e*** is the random residuals and is set as $\boldsymbol e\sim\mathbf N\left(\mathbf0,\boldsymbol I\boldsymbol\sigma_{\boldsymbol e}^{\mathbf2}\right)$, where ***I*** is an identity matrix. The analyses were performed using GEMMA software [18]. The Bonferroni correction was applied to filter the potential SNPs: SNPs with permuted *P*-values lower or equal than 0.05/N (N is the number of the independent markers defined as a set of SNPs with pairwise r square value higher than 0.4), were regarded as genome-wide significant SNPs. SNPs with a *P*-value higher than 0.05/N but lower than 1/N were considered as genome-wide suggestive significant SNPs. Then, quantile-quantile (Q-Q) plots were drawn and the inflation factors (λ) were calculated to check the population stratification.

In order to uncover pleiotropic effects of the intron mutation (rs346331089) and its potential effect on the phenotype in Duroc pigs, we utilized our previous reported data sets of economic traits, followed by implementing the association analyses between rs346331089 and economic traits using PLINK with “-linear” parameter.

### Tissue-specific genes and pCADD scores annotation

We downloaded a gene expression matrix of Duroc pig tissues (i.e., fat, heart, liver, muscle, spleen, cerebellum, cerebrum, duodenum, kidney, lung, thymus) from publicly available datasets [19]. As in Zhao et al. [19], genes with an expression level at least three times higher in a given tissue than in any other tissue were considered to be tissue-specific. Meanwhile, we selected human-pig homologous genes from Ensembl release 105 and used the human protein atlas [20] to further explore the expression of human-pig homologous genes with high confidence. Furthermore, pCADD scores were retrieved from public databases [21] to prioritize variants.

### Functional enrichment analysis

The Animal QTL Database [22] was used to annotate the potential functions of the selection regions. QTL enrichment analyses based on a bootstrap simulation for each QTL were conducted to annotate selection signatures, and the adjusted *P*-value based on multiple tests less than 0.05 were retained. Furthermore, the genes located in putative selection regions were identified using R package GALLO [23]. Then, the positional candidate genes overlapped with the genomic regions for NT traits were extracted based on *Sus scrofa* 11.1 reference genome assembly. We used R package clusterProfiler [24] to conduct enrichment analyses, then the Kyoto Encyclopedia of Genes and Genomes (KEGG) pathways and GO terms with Benjamini-Hochberg method adjusted *P*-value < 0.05 were selected.

## Results

### Whole-genome sequencing and imputation

The genomic DNA extracted from key individuals (*n* = 50) of a factory-farmed Duroc pig population was re-sequenced with 150 bp paired-end reads using the Illumina HiSeq 3000 platform. A total of 19,754,293 SNPs was found, of which 6.13% were novel in comparison with the latest pig SNP database (European variant archive; updated time: 24-Feb-2022). In addition, transition-to-transversion (TS/TV) ratios of SNPs was 2.33. With the use of Variant Effect Predictor (VEP) to determine the effect of the variants, we obtained location of the variants and the most severe consequence of the variants on the protein sequence. The most SNPs (59.55% of all SNPs) were located in intron regions, followed by intergenic (19.94%), downstream genic (8.71%), and upstream genic regions (8.37%). Moreover, the plot showed that SNPs were evenly distributed across porcine autosomes (Fig. S1).

After imputation with key individuals as reference panel, we obtained a large-scale genotyping dataset contained 8,766,074 high-quality SNPs with dosage R-squared (DR^2^) > 0.8. The proportion of each consequence types was broadly similar with reference panel, and the distribution of SNPs was shown in Fig. S2. Therefore, a total of 1067 Duroc pigs with 8,766,074 SNPs were available for further analyses.

### Identification of human-induced polygenic selection

**Fig. 1:**
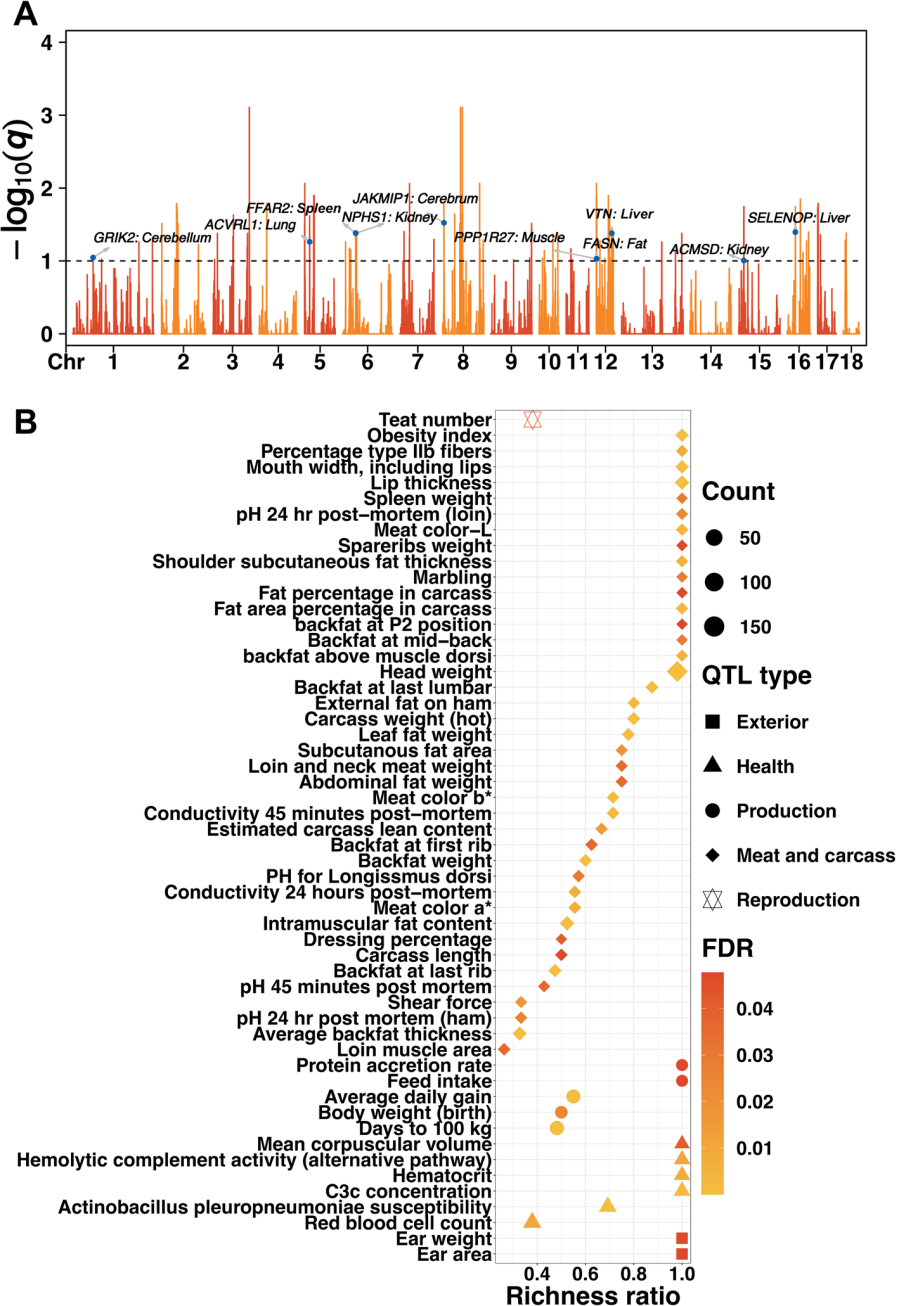
GPSM detects signatures of ongoing polygenic selection in the Chinese Duroc pigs. **A** GPSM Manhattan plots for the Chinese Duroc pigs. The Y-axis displayed the -log_10_(*q*) of SNP according to their chromosomal position (X-axis). The horizontal dashed line depicts the genome-wide significance level (*q*-value < 0.1). The significant variants were annotated using tissue-specific genes. **B** QTL enrichment analyses with GPSM signals. The richness factor was obtained by the ratio of the number of QTLs annotated in the candidate regions and the total number of each QTL

Based on results from the GPSM analyses (Fig. 1A), 5649 SNPs (*q*-values < 0.1) were significantly associated with birth date in the Chinese Duroc pig population (Table S2). We explored the potentially biological function of detected GPSM signals with genome annotation, e.g., tissue-specific genes and publicly available QTLs.

Twenty-two out of 24 tissue-specific genes covered by GPSM signals were homologous between pig and human with high confidence, and a majority of these genes had similar expression patterns with tissue specificity in human (Table 1). Further, we found that the expression levels of cerebellum, cerebrum, and fat tissue-specific genes covered by GPSM signals were the most conserved between pig and human. The strongest association signals were located in *Sus scrofa* chromosome (SSC) 3 (Fig. S3A) and 8 (Fig. S3B), we observed high LD between the “lead” SNP and SNPs around the “lead” SNP. QTL enrichment analyses with the signals showed that production, meat and carcass traits were mostly significantly enriched (Fig. 1B). Interestingly, we noticed that “Teat number” QTL was also significantly enriched.

**Table 1:** Tissue-specific genes that overlapped with the genome-wide significant GPSM signals

Chr^**a**^	Position	SNP	***P***-value	Gene^**b**^	Description	Tissue^**c**^	Type of orthologue^**d**^
1	68,477,634	rs343429147	4.99 × 10^−5^	*GRIK2*	Glutamate ionotropic receptor kainate type subunit 2	Cerebellum	1-to-1*
5	17,275,197	rs325589813	2.22 × 10^−5^	*ACVRL1*	Activin A receptor like type 1	Lung	1-to-1*
6	44,902,082	rs333577047	9.06 × 10^−6^	*FFAR2*	Free fatty acid receptor 2	Spleen	1-to-1*
6	45,249,022	6_45249022	1.34 × 10^−5^	*NPHS1*	NPHS1 adhesion molecule, nephrin	Kidney	1-to-1*
8	4,497,067	rs325024132	2.96 × 10^−6^	*JAKMIP1*	Janus kinase and microtubule interacting protein 1	Cerebrum	1-to-1*
8	126,046,420	rs325782763	3.11 × 10^−5^	*GRID2*	Glutamate ionotropic receptor delta type subunit 2	Cerebellum	1-to-1*
12	921,691	rs321539153	5.40 × 10^−5^	*FASN*	Fatty acid synthase	Fat	1-to-1
12	1,136,822	12_1136822	5.40 × 10^−5^	*PPP1R27*	Protein phosphatase 1 regulatory subunit 27	Muscle	1-to-1*
12	44,658,312	rs694026589	5.71 × 10^−5^	*VTN*	Vitronectin	Liver	1-to-many*
12	44,734,028	rs328927732	1.99 × 10^−5^	*SLC13A2*	Solute carrier family 13 member 2	Duodenum	1-to-1*
12	54,906,956	rs329254243	2.18 × 10^−5^	*GAS7*	Growth arrest specific 7	Cerebrum	1-to-1*
15	17,161,987	rs322661315	6.29 × 10^−5^	*ACMSD*	Aminocarboxymuconate semialdehyde decarboxylase	Kidney	1-to-1*
16	27,537,893	rs335570719	6.74 × 10^−6^	*SELENOP*	Selenoprotein P	Liver	1-to-1*
17	2,034,960	17_2034960	2.72 × 10^−5^	*SGCZ*	Sarcoglycan zeta	Cerebrum	1-to-1*
17	9,824,048	rs331351838	4.92 × 10^−5^	*ZMAT4*	Zinc finger matrin-type 4	Cerebrum	1-to-1*

^a^*Sus scrofa* chromosome

^b^The promising candidate genes

^c^Specific expression tissues

^d^A type of orthologue assigned for *sus scrofa* and *homo sapiens*

To further estimate the direction of selection on NT relevant traits, we summarized the phenotypic records and found that there were significant differences in the number of teats between pigs born on 2009 and 2017 (a Welch Two Sample *t*-test *P*-value = 0.0039). Moreover, we conducted $\hat{G}$ analyses with phenotypic records, and observed significant evidence of selection for decreased total teat number (*P*-value = 0.0002, Fig. S4A), left teat (*P*-value = 0.0003, Fig. S4B), and right teat (*P*-value = 0.0009, Fig. S4C). These results uncovered that NT relevant traits had been sufficiently human-induced selected in the past few years in the Chinese Duroc pig population.

The genome-wide ROH were assessed on autosomes, and a sum of 152,961 ROH were detected in the studied population (Fig. S5A). In addition, a total of 69 candidate genes were overlapped with ROH hotspots (Table S3). Although we did not identify the common candidate genes between the GPSM analyses and ROH hotspots detection, the QTL enrichment results showed that ROH hotspots were also mostly enriched in meat and carcass traits (Fig. S5B).

### Teat number relevant traits-specific selection signature detection

**Fig. 2:**
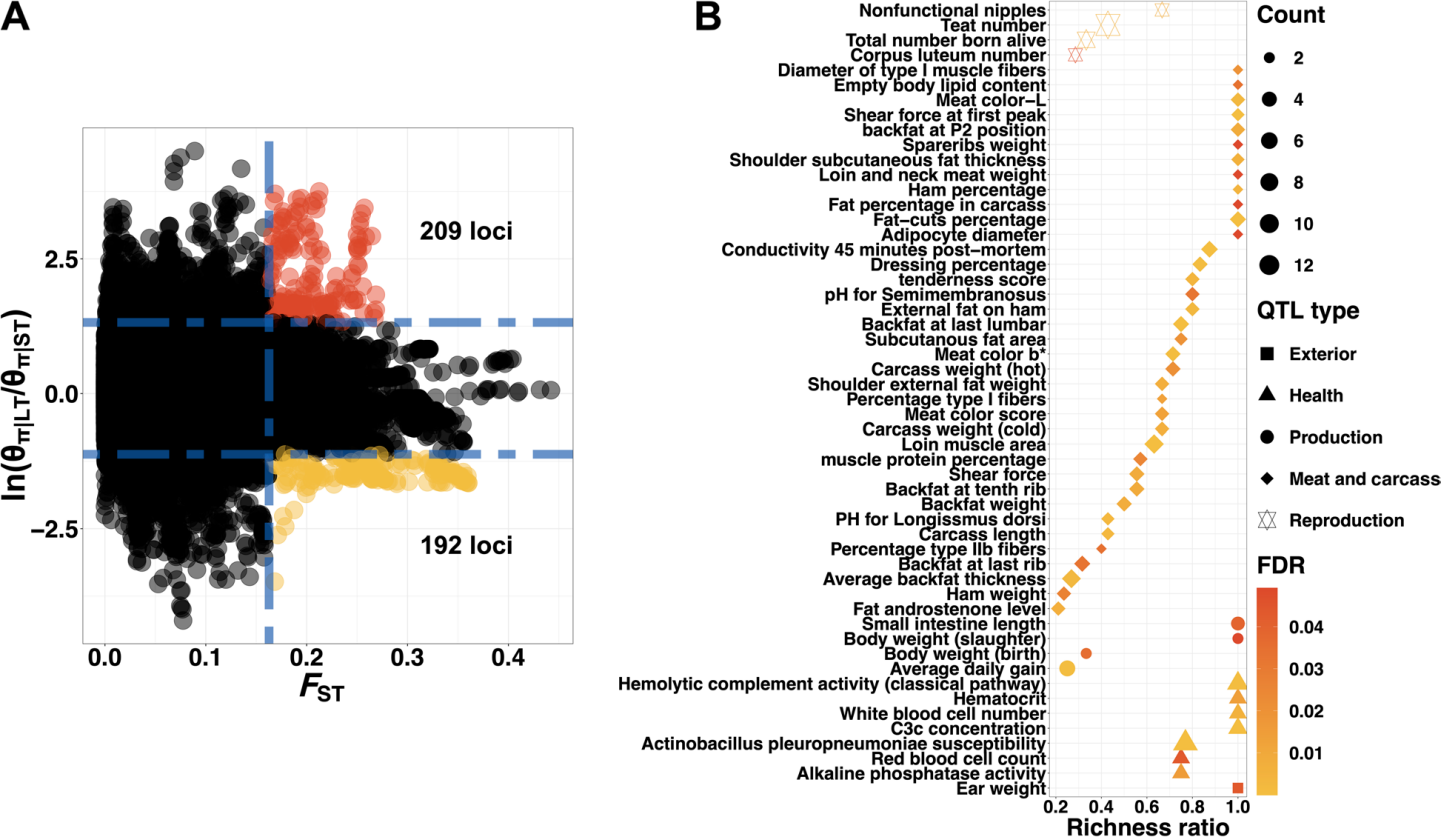
Teat number relevant traits-specific selection signature detection. **A** Detection of traits-specific selection signatures based on the constructed population pairs with extreme differences in total teat number. The Y-axis displayed the θ_π_ ratio and the X-axis showed the *F*_ST_ value. The 1% of windows with the highest θ_π_ ratio and *F*_ST_ values was considered the potential selection regions. **B** QTL enrichment analyses with teat number relevant traits-specific selection signature detection. The richness factor was obtained by the ratio of the number of QTLs annotated in the candidate regions and the total number of each QTL

We conducted detection of traits-specific selection signatures based on the constructed population pairs with extreme differences in total teat number. Here, we detected 401 positive selection signatures (Fig. 2A), of which 209 loci reflected the loss of nucleotide diversity in LT group relative to ST group, while 192 loci were opposite. QTLs enrichment analyses showed that “teat number” QTL was indeed enriched, meanwhile, we noticed that the selection signatures significantly overlapped with several QTLs related to production, meat and carcass traits (Fig. 2B).

A total of 78 candidate genes were overlapped with NT relevant traits-specific selection signatures, and 13 out of these genes showed tissue-specific expression levels. Here, we found that *ASPH*, *CCDC85C*, *CYP46A1*, *ASGR2*, *PNLIP*, *PNLIPRP1*, *DPP10*, EPHA4*,* and *PLCB4* had similar RNA tissue specificity in pig and human (Table 2). Functional annotations were significantly enriched in lipid metabolism related pathways and terms, e.g., “pancreatic secretion”, “fat digestion and absorption”, “glycerolipid metabolism”, and “lipid catabolic process”. These results proved that the trait-specific selection signatures were mainly caused by phenotypic difference and uncovered that NT relevant traits-related candidate genes played potential roles in lipid metabolism.

**Table 2:** Tissue-specific genes that overlapped with teat number relevant traits-specific selection signature

Chr^**a**^	Start^**b**^	End^**c**^	Gene^**d**^	Description	Tissue^**e**^	Type of orthologue^**f**^
4	71,960,001	72,010,000	*ASPH*	Aspartate beta-hydroxylase	Fat	1-to-1*
7	120,550,001	120,600,000	*CCDC85C*	Coiled-coil domain containing 85C	Cerebellum	1-to-1*
7	120,650,001	120,700,000	*CYP46A1*	Cytochrome P450 family 46 subfamily A member 1	Cerebrum	1-to-1*
12	52,430,001	52,480,000	*ASGR2*	Asialoglycoprotein receptor 2	Liver	1-to-1*
14	126,740,001	126,820,000	*PNLIP*	Pancreatic lipase	Duodenum	1-to-1*
14	126,740,001	126,820,000	*PNLIPRP1*	Pancreatic lipase related protein 1	Duodenum	1-to-1*
15	20,730,001	20,780,000	*DPP10*	Dipeptidyl peptidase like 10	Cerebrum	1-to-1*
15	123,440,001	123,490,000	*EPHA4*	EPH receptor A4	Cerebrum	1-to-1*
17	18,010,001	18,060,000	*PLCB4*	Phospholipase C beta 4	Cerebellum	1-to-1*

^a^*Sus scrofa* chromosome

^b^The start of the potential selection regions

^c^The end of the potential selection regions

^d^The promising candidate genes

^e^Specific expression tissues

^f^A type of orthologue assigned for *sus scrofa* and *homo sapiens*

### Imputed sequence-based GWAS for teat number relevant traits

**Fig. 3:**
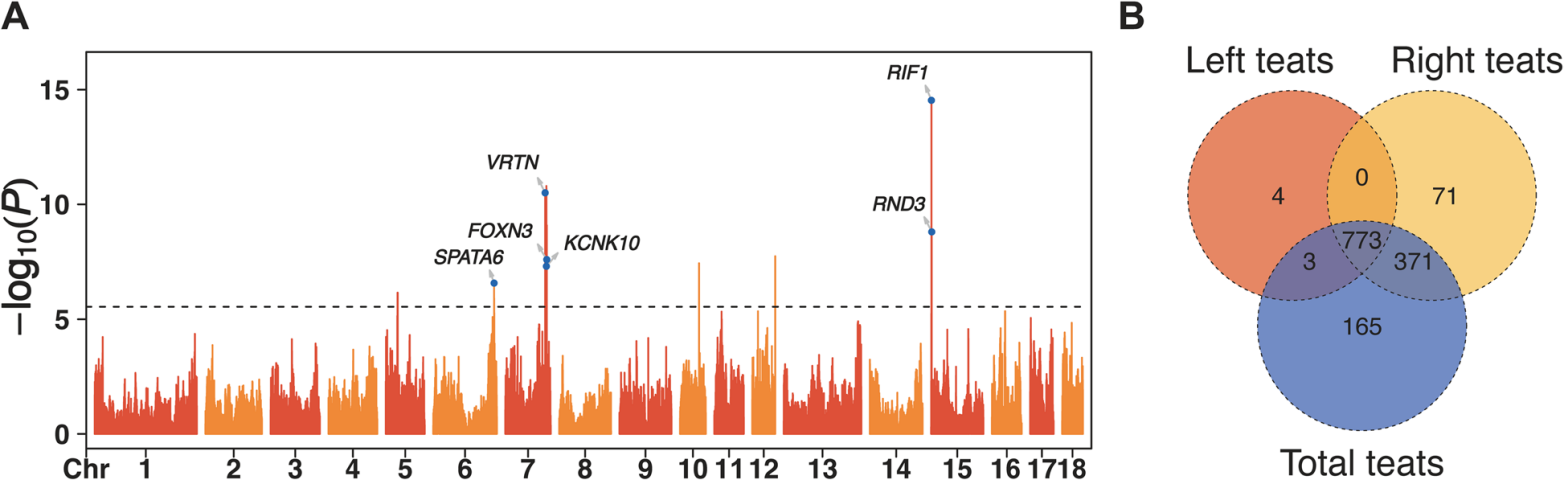
The Manhattan plots of GWAS for the number of total teats (NT) traits. **A** The Y-axis of Manhattan plots displayed the -log_10_(*P*) of each SNP in the genome wide association analysis for NT traits, the X-axis represented the position of SNPs for chromosomes. **B** The Venn diagram of the putative loci that associated with the number of left teats, right teats, and total teats

Using the number of left teats, right teats, and total teats as phenotypic records, a total of 1387 putative loci were significantly associated with NT relevant traits, of which 4, 71, and 165 putative loci were uniquely detected in imputed sequence-based GWAS for the number of left teats (Fig. S6A), right teats (Fig. S6B), and total teats (Fig. 3A), respectively. Meanwhile, 773 of all putative loci were shared by GWAS for these three NT relevant traits (Fig. 3B). *SPATA6*, *VRTN*, *FOXN3*, *KCNK10*, *RND3*, and *RIF1* were covered and identified as the promising candidate underlying NT relevant traits. We found that some of the promising candidate genes were also associated with fat-related traits, e.g., *KCNK10* and *RND3*, which was consistent with the results of QTL enrichment analyses (Fig. S6C).

### Comparison between traits-specific selection signature and GWAS

**Fig. 4:**
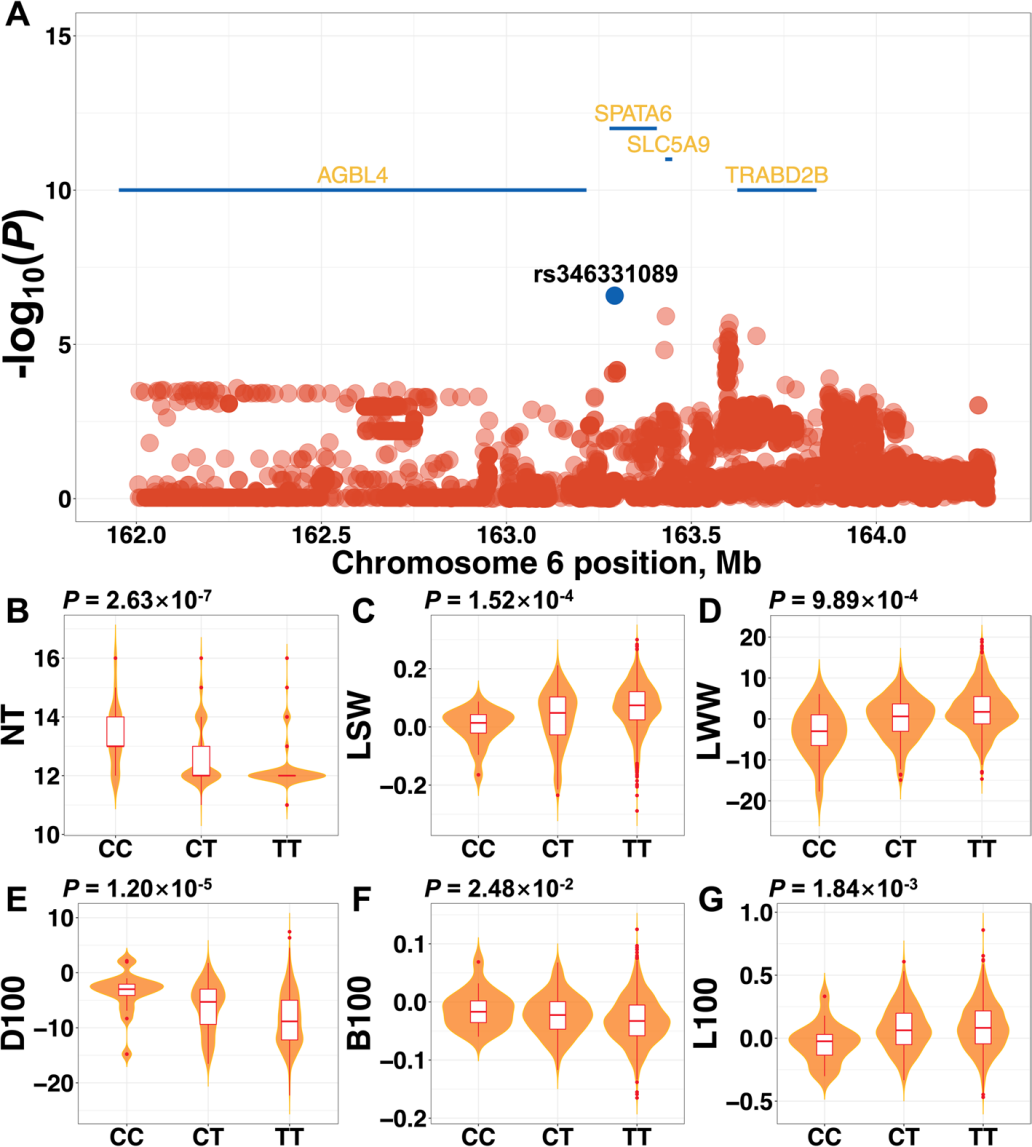
The pleiotropic variant rs346331089 and its effects on economic traits. **A** Regional association plots around rs346331089. Genotype effect plots of rs346331089 among three types for teat number (**B**), litter size at weaning (**C**), litter weight at weaning (**D**), days to 100 kg (**E**), backfat thickness at 100 kg (**F**), and loin muscle area at 100 kg (**G**)

Combining results of NT relevant traits-specific selection signatures and GWAS, we found three common genome regions, located in SSC 6, 7, and 15, respectively. In these regions, rs346331089 (Fig. 4), rs322980623 (Fig. S7), and rs324534752 (Fig. S8) got the highest pCADD score. The regions located in SSC6 significantly enriched in the QTLs related to litter size, rump width, oleic acid content, and top line conformation traits (Fig. S9A), the regions located in SSC7 were associated with obesity index, head weight, mean corpuscular volume, mean corpuscular hemoglobin concentration, and cannon bone circumference traits (Fig. S9B), and the regions located in SSC 15 overlapped with QTLs related to backfat between 3nd and 4th last ribs and hematocrit traits (Fig. S9C). These results suggested that there were a few pleiotropic genes in these genome regions, influencing both NT and economic traits.

### Identification of pleiotropic variants underlying teat number and economic traits

As one of the most utilized commercial composite boar lines, it is well known that Duroc pigs had been under selection for production and meat quality traits. Nevertheless, as mentioned above, NT relevant traits have experienced human-induced selection in the Chinese Duroc pig population. We assumed that a few pleiotropic variants were putative contradictive loci that played an opposite directional role in teat number and economic traits, leading to decreased left teat, right teat, and total teat number.

We further found that rs346331089 has pleiotropic gene action on NT and economic traits, e.g., LSW, LWW, D100, B100, and L100 traits (Fig. 4**)**. Genotype effects of rs346331089 on NT traits showed similar trends in its effects on D100 and B100 traits, whereas its effects had opposite patterns for LSW, LWW, and L100 traits. Overall, human-induced selection for the genetic improvement (e.g., faster growth rates, thinner backfat thickness, and larger loin muscle area) at the genome regions might lead to the decreasing number of teats.

## Discussion

In this study, we detected polygenic selection in a factory-farmed Duroc pigs. Genes related to teat relevant traits were then dissected by combined analyses of selection signatures and GWAS. Pleiotropic variants underlying teat number and economic traits were further confirmed.

### Human-induced polygenic selection in Duroc pigs

Based on the results of the GPSM analyses, we infer that the Chinese Duroc pig population was mainly subjected to high-intensity artificial selection on production and meat quality traits, in line with the breeding goals of Duroc pigs [25]. Further gene annotations showed a set of candidate genes involved in artificial selection, e.g., *GRIK2*, *JAKMIP1*, *GRID2*, *FASN*, *GAS7*, *SELENOP*, and *SGCZ*. *GRIK2* belongs to the kainate family of glutamate receptors. Previous study suggested that *GRIK2* played a role in affecting intermuscular fat level [26]. We observed the frequencies of *GRIK2* mutations were obviously different between the population in 2009 and 2017, these mutations might affect the normal transcription and expression of *GRIK2* and further have impacts on intermuscular fat level, which is an important meat quality parameter. Also, *JAKMIP1* involved in the actions of neurons, which are central regulators in maintaining the balance between food intake and energy expenditure, and further regulated fat deposition in muscle [27]. *FASN* is related to lipogenesis and has been found to have potential roles in the determination of feed conversion and meat color in pigs [28]. *GAS7* was implicated in influencing the fatty acid composition of the longissimus dorsi muscle in pigs [29]. Moreover, *SGCZ* has potential functions in fat deposition in chicken [30]. In addition, a few genes (e.g., *GRID2* and *SELENOP*) were reported to be associated with reproduction traits [31, 32].

In addition, we detected ROH hotspots to explore the selection landscape of the studied population. A set of candidate genes (e.g., *MAD2L1* and *SEC24D*) that were putatively under selection in American and Canadian Duroc pigs have been previously reported [33]. Likewise, we found that the candidate genes were vastly different between the GPSM analyses and ROH hotspots detection. This might be caused by the different approaches: the GPSM analyses detected ongoing selection in terms of heterozygosity, and ROH hotspots detection identified selection in terms of regions of homozygosity. Therefore, the findings would be more comprehensive by combining the results of the GPSM analyses with ROH hotspots detection. Interestingly, we observed that the GPSM signals and ROH hotspots were both mostly significantly enriched in production, meat and carcass traits, as other traits were rarely enriched. Altogether, genetic improvement of Duroc pigs in China through selection on genes that are correlated with economic characters (e.g., production and meat quality) has been mainly considered during artificial selection.

### Traits-specific selection signature and GWAS for teat number relevant traits

During the rapid improvement of the performance of economic traits, the number of teats has decreased in the Chinese Duroc pig population. We hypothesized that genetic correlations between NT and economic traits. Traits-specific selection signatures were high enriched in both teat number relevant traits and fat-related traits, which confirmed that the hypothesis was correct. Moreover, several candidate genes (e.g., *ASPH*, *CYP46A1*, *PNLIP*, *PNLIPRP1*, *DPP10*, *EPHA4*, and *PLCB4*) overlapped with traits-specific selection signatures have underlying correlations with economically significant traits. *ASPH* are involved with tissue morphology, skeletal and muscle development, and fat deposition [34]. Also, *CYP46A1*, *PNLIP*, and *DPP10* have been identified as regulators of lipid metabolism [35–37]. *EPHA4*, which was detected in the endometrium during embryo implantation in pigs, was found to have relationships with reproduction traits [38]. Moreover, *PLCB4* was implicated in growth and stature traits, and has been identified as genes under directional selection between Duroc and Duroc synthetic pig populations [39]. Further, we focused on detection of candidate genes that related to NT relevant traits. Interestingly, several genes located on the region ranged from 110.13 Mb to 110.47 Mb on chromosome 7 were found, and also reported in previous GWAS for NT trait in pigs [40]. Among these positional candidate genes, *KCNK10*, as a member of tandem pore domain potassium channel family, involves in stabilizing the negative resting membrane potential and counterbalancing depolarization. *KCNK10* has been reported that it is a regulator of mitotic clonal expansion during the adipocyte differentiation [41]. *FOXN3* could be considered as a candidate gene for the hairless phenotype in pigs [42]. In addition, *RND3* was identified as a candidate gene for residual feed intake in pigs [43] and *RIF1* was one of putative regulatory factors that contribute to the molecular mechanisms that underlie fat content and energy balance in muscle [44]. Overall, these results implied that there were a few pleiotropic variants underlying teat number and economic traits (e.g., growth, fatness, and reproduction traits).

### Pleiotropic variants underlying teat number and economic traits

In addition, we found that genotype effects of rs346331089 on NT reflected similar trends with D100 and B100 traits, whereas had opposite trends with LSW, LWW, and L100 traits. The variant rs346331089 located in the intron of *SPATA6*, which is one of sperm-specific genes. Previous studies suggested the regulation of the expression pattern of *SPATA6* linked to spermatogenesis in Hu sheep [45] and inactivation of *SPATA6* leaded to acephalic spermatozoa and male sterility in mice [46]. These results above manifested high-intensity directional selection on certain economic traits might influenced the number of teats in pigs.

## Conclusions

In this study, we detected polygenic selection in a factory-farmed Duroc pigs and dissected the candidate genes related to teat number relevant traits by combined analyses of selection signatures and GWAS. The variant rs346331089 has pleiotropic effects on teat number relevant traits and economic traits. Our findings showed that genetic improvement through human-induced selection on genes that are correlated with economically important traits may lead to the decreasing number of teats, and contributed to guide the further breeding of Duroc pigs.

## Supplementary Information

**Additional file 1: Fig. S1.** The distribution (A) and functional classification (B) of the detected single-nucleotide polymorphisms (SNPs).

**Additional file 2: Fig. S2.** The distribution (A) and functional classification (B) of the Imputed single-nucleotide polymorphisms (SNPs).

**Additional file 3: Fig. S3.** The LD block in significant chromosome regions located in *Sus scrofa* chromosome (SSC) 3 (A) and 8 (B).

**Additional file 4: Fig. S4.** Ghat analysis with phenotypic records of the number of total teats (A), left teats (B), and right teats (C).

**Additional file 5: Fig. S5.** Detection of ROH in the Chinese Duroc pig population. (A) Manhattan plot of the occurrence (%) of each SNP in ROHs of Duroc pigs. The dashed line corresponds to the significance threshold. (B) QTL enrichment analyses with ROH hotspots. The richness factor was obtained by the ratio of the number of QTLs annotated in the candidate regions and the total number of each QTL.

**Additional file 6: Fig. S6.** The Manhattan plots of GWAS for the number of left and right teats traits. The Y-axis of Manhattan plots displayed the -log_10_(P) of each SNP in the genome wide association analysis for the number of left (A) and right teats (B) traits, the X-axis represented the position of SNPs for chromosomes. (C) QTL enrichment results of GWAS on teat number relevant traits.

**Additional file 7: Fig. S7.** Regional association plots around rs322980623. (A) GWAS result around rs322980623. (B) pCADD values around rs322980623.

**Additional file 8: Fig. S8.** Regional association plots around rs324534752. (A) GWAS result around rs324534752. (B) pCADD values around rs324534752.

**Additional file 9: Fig. S9.** QTL enrichment results of the potential selection regions around rs346331089 (A), rs322980623 (B), and rs324534752 (C).

**Additional file 10: Table S1.** The pairwise difference of teats number between each birth year using a Welch Two Sample t-test.

**Additional file 11: Table S2.** The significant Generation Proxy Selection Mapping (GPSM) signals.

**Additional file 12: Table S3.** Candidate genes that overlapped with ROH hotspots.

**Additional file 13: Table S4.** Candidate genes that overlapped with teat number relevant traits-specific selection signature.

## Data Availability

The datasets used and analyzed during this study are available from the corresponding author upon reasonable request.

## Abbreviations

GPSM: Generation Proxy Selection Mapping
NT: Teat number
LSW: Litter size at weaning
LWW: Litter weight at weaning
D100: Days to 100 kg
B100: Backfat thickness at 100 kg
L100: Loin muscle area at 100 kg
QTL: Quantitative trait loci
GWAS: Genome-wide association study
***G***: Genomic relationship
LD: Linkage disequilibrium
MAF: Minor allele frequency
SNP: Single nucleotide polymorphism
WGS: Whole genome sequencing
