# Supplementary figures and images for: Insights into the architecture of human-induced polygenic selection in Duroc pigs
Source: J Anim Sci Biotechnol. 2022 Sep 21;13:99. doi: 10.1186/s40104-022-00751-x (PMC9490910; doi:10.1186/s40104-022-00751-x)

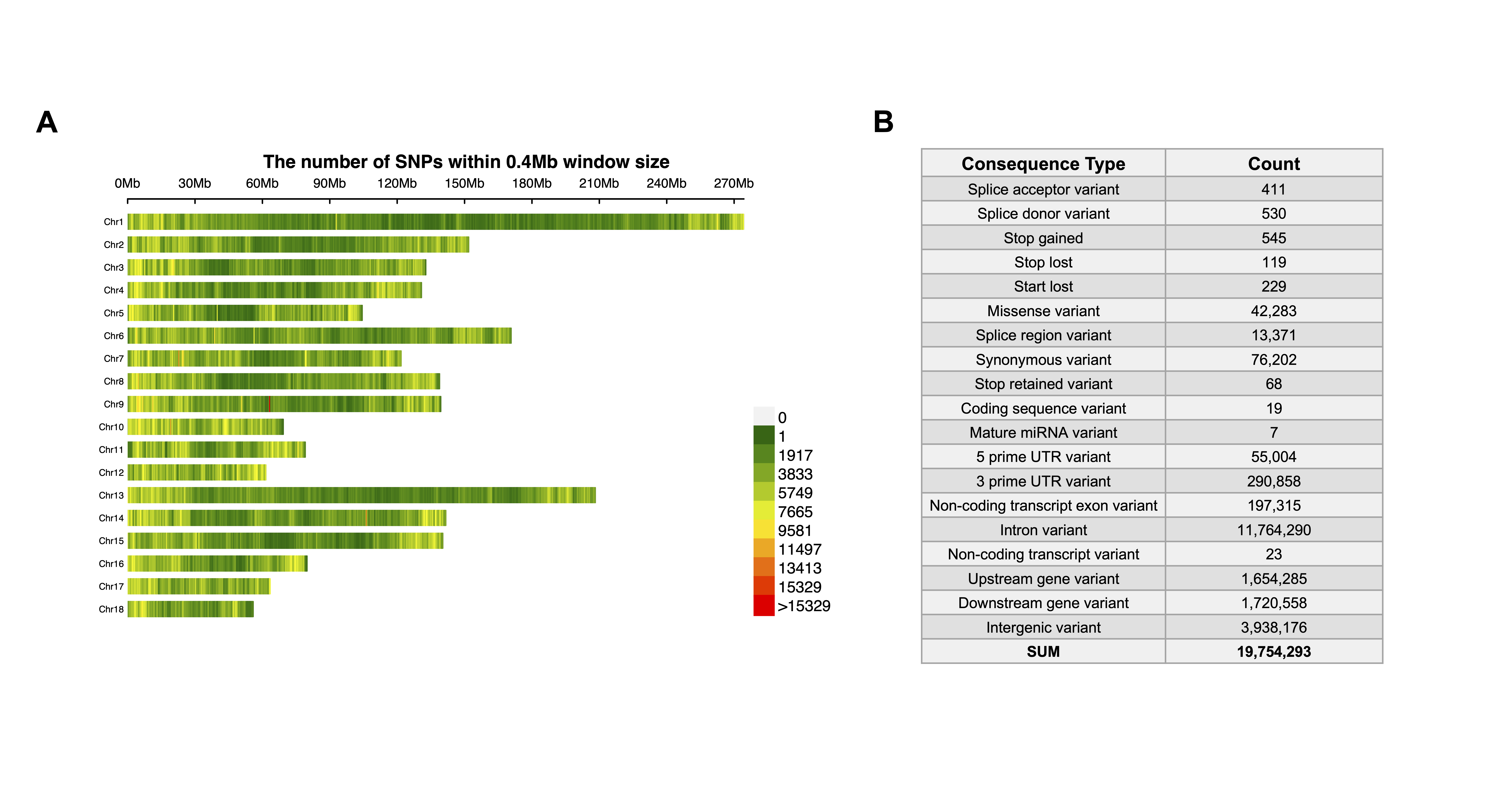

Supplement: Supplementary file 1 — Additional file 1: Fig. S1. The distribution (A) and functional classification (B) of the detected single-nucleotide polymorphisms (SNPs). [file 40104_2022_751_MOESM1_ESM.png]

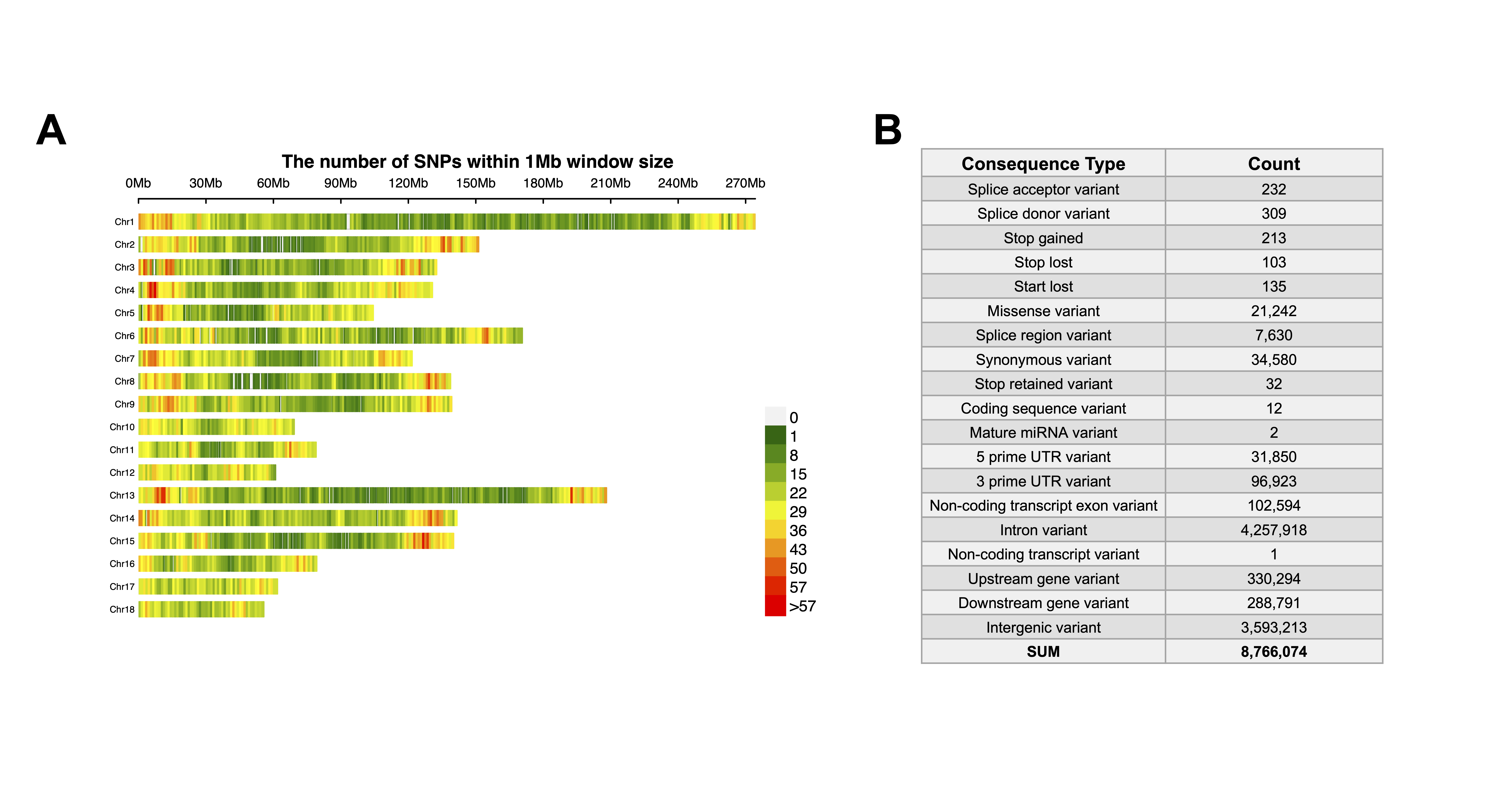

Supplement: Supplementary file 2 — Additional file 2: Fig. S2. The distribution (A) and functional classification (B) of the Imputed single-nucleotide polymorphisms (SNPs). [file 40104_2022_751_MOESM2_ESM.png]

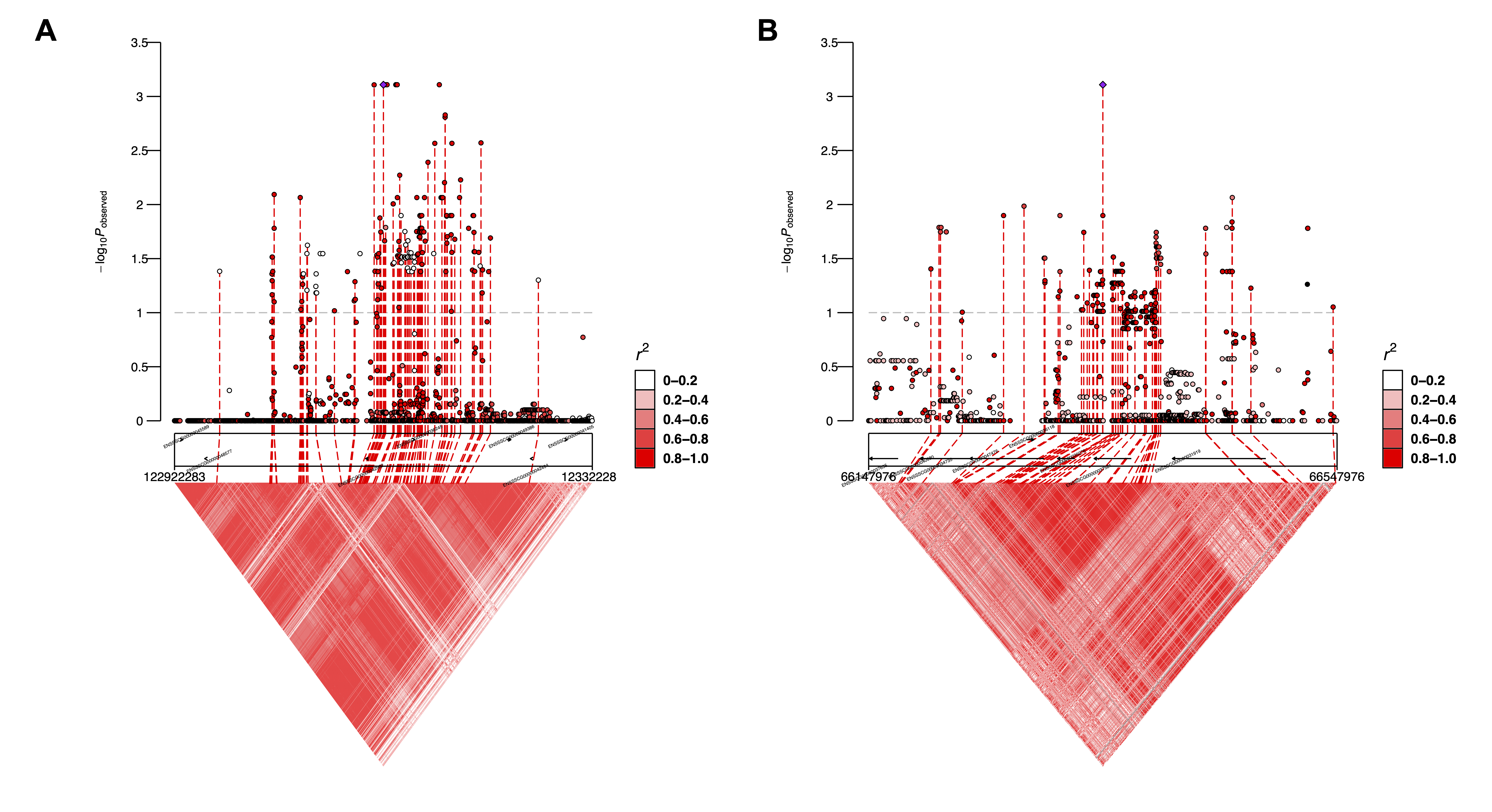

Supplement: Supplementary file 3 — Additional file 3: Fig. S3. The LD block in significant chromosome regions located in Sus scrofa chromosome (SSC) 3 (A) and 8 (B). [file 40104_2022_751_MOESM3_ESM.png]

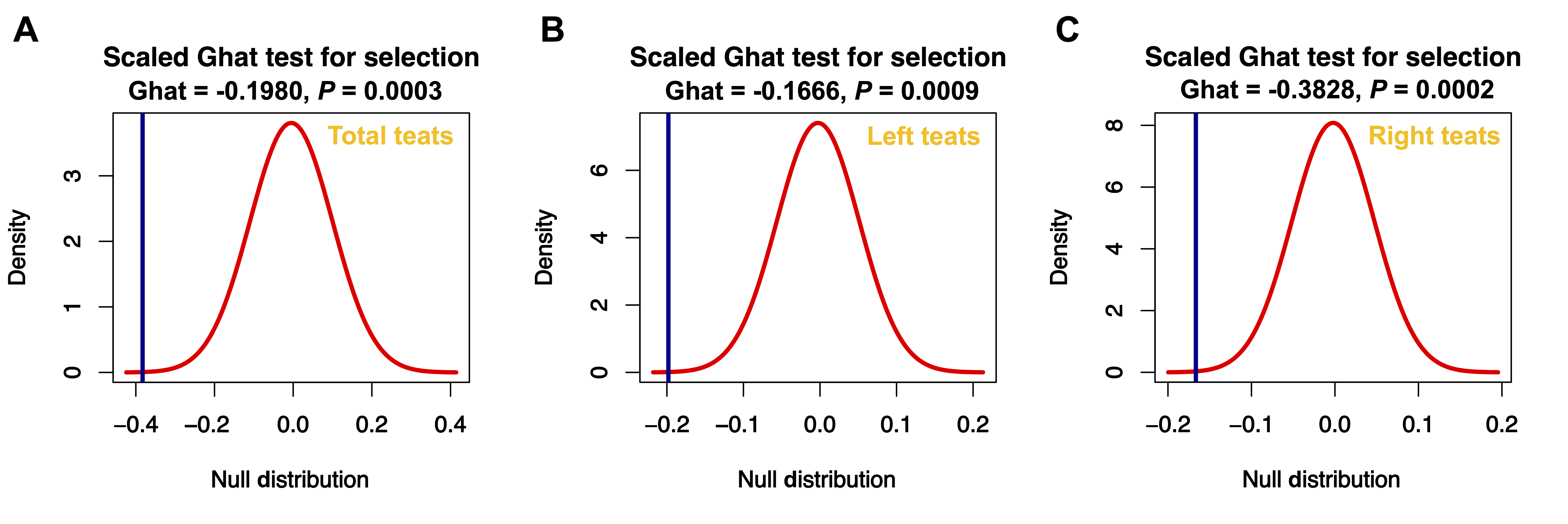

Supplement: Supplementary file 4 — Additional file 4: Fig. S4. Ghat analysis with phenotypic records of the number of total teats (A), left teats (B), and right teats (C). [file 40104_2022_751_MOESM4_ESM.png]

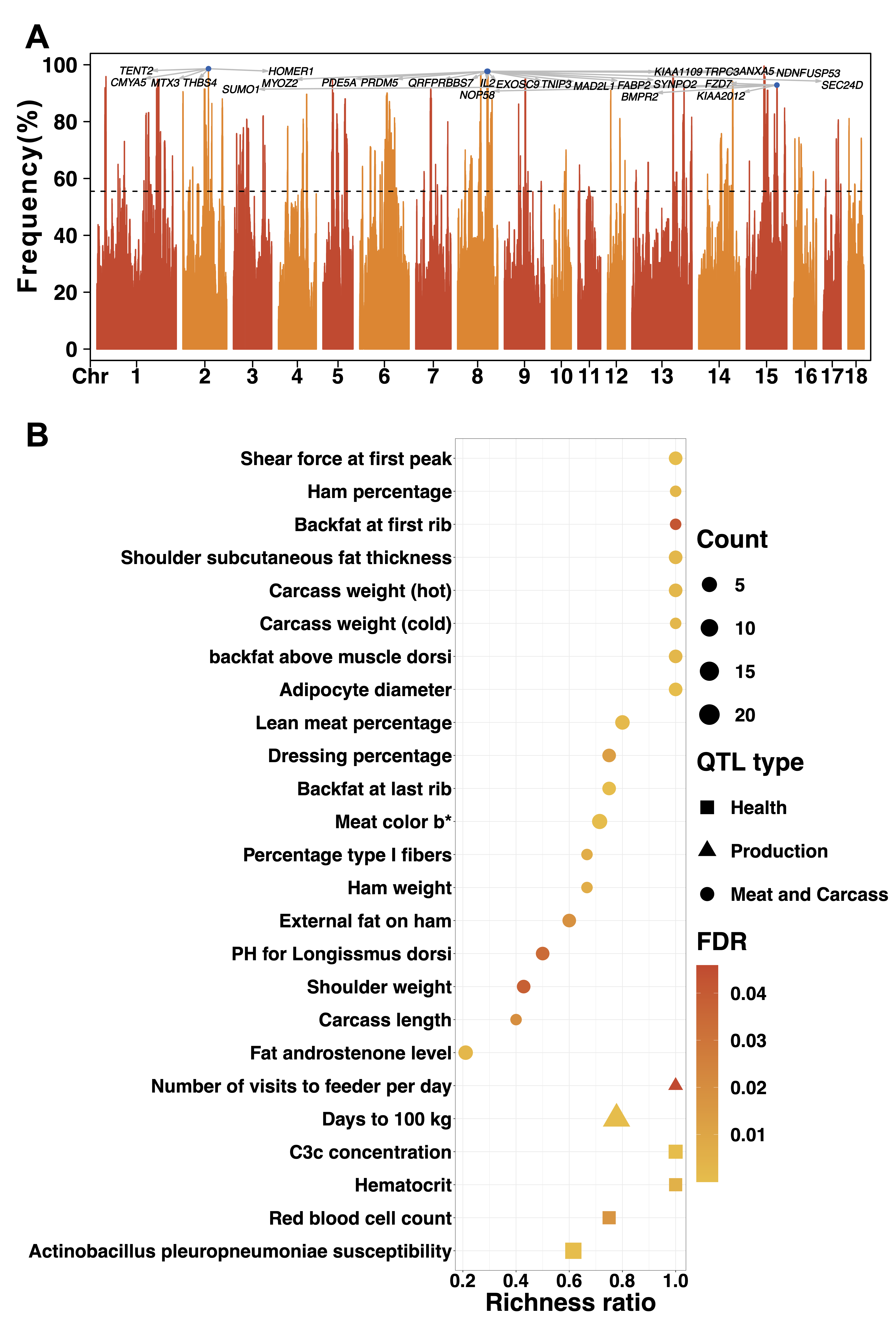

Supplement: Supplementary file 5 — Additional file 5: Fig. S5. Detection of ROH in the Chinese Duroc pig population. (A) Manhattan plot of the occurrence (%) of each SNP in ROHs of Duroc pigs. The dashed line corresponds to the significance threshold. (B) QTL enrichment analyses with ROH hotspots. The richness factor was obtained by the ratio of the number of QTLs annotated in the candidate regions and the total number of each QTL. [file 40104_2022_751_MOESM5_ESM.png]

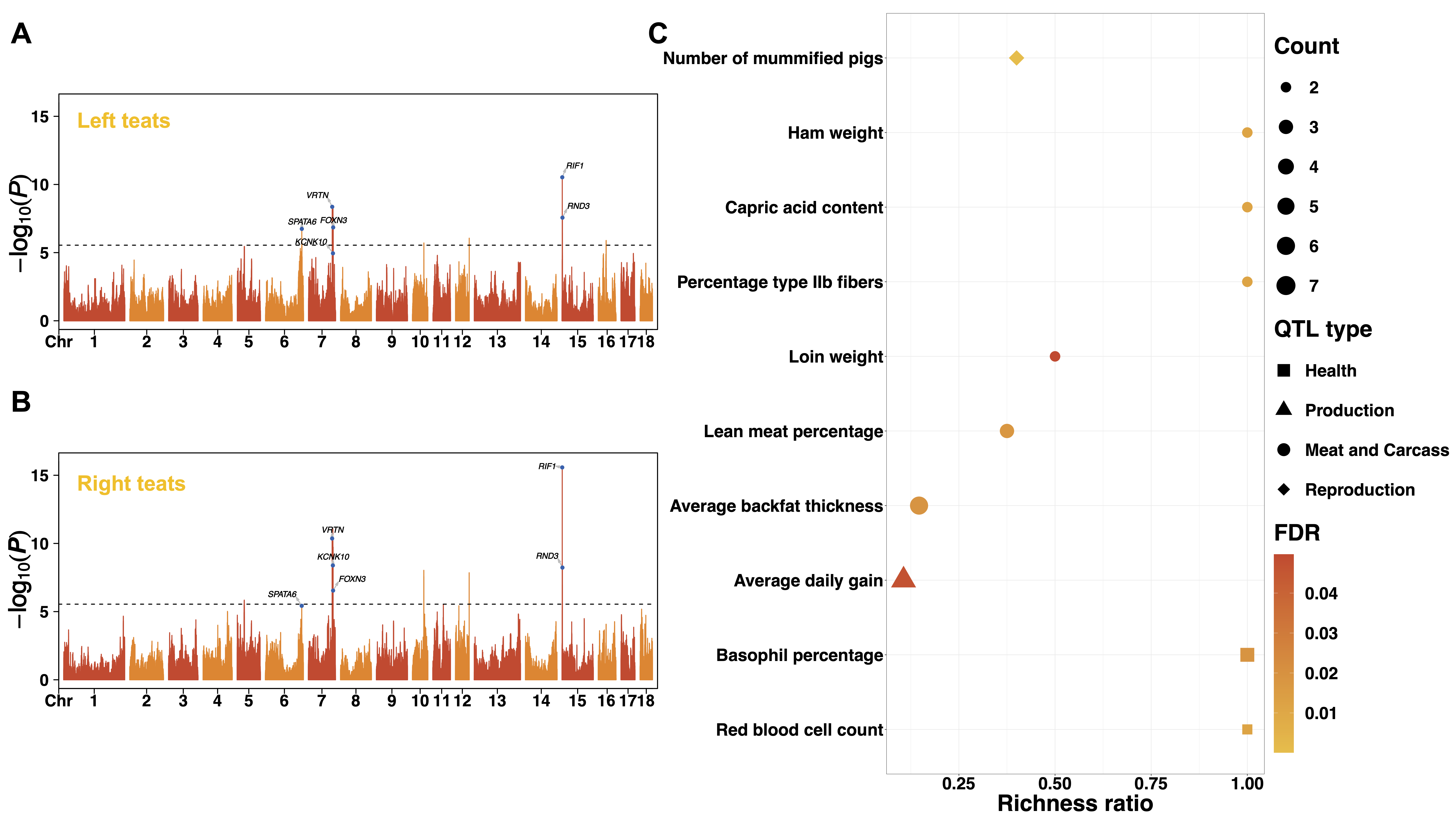

Supplement: Supplementary file 6 — Additional file 6: Fig. S6. The Manhattan plots of GWAS for the number of left and right teats traits. The Y-axis of Manhattan plots displayed the -log10(P) of each SNP in the genome wide association analysis for the number of left (A) and right teats (B) traits, the X-axis represented the position of SNPs for chromosomes. (C) QTL enrichment results of GWAS on teat number relevant traits. [file 40104_2022_751_MOESM6_ESM.png]

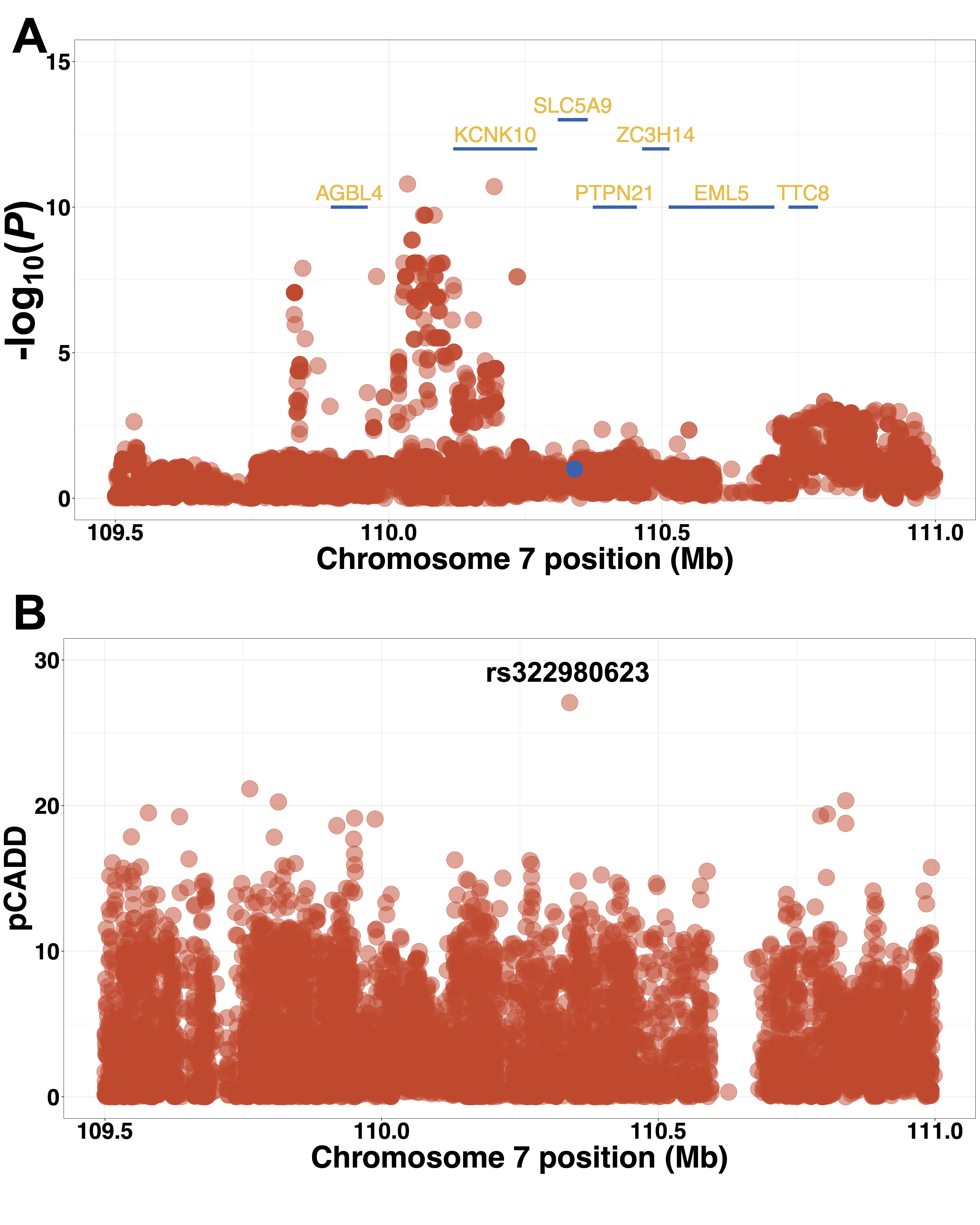

Supplement: Supplementary file 7 — Additional file 7: Fig. S7. Regional association plots around rs322980623. (A) GWAS result around rs322980623. (B) pCADD values around rs322980623. [file 40104_2022_751_MOESM7_ESM.png]

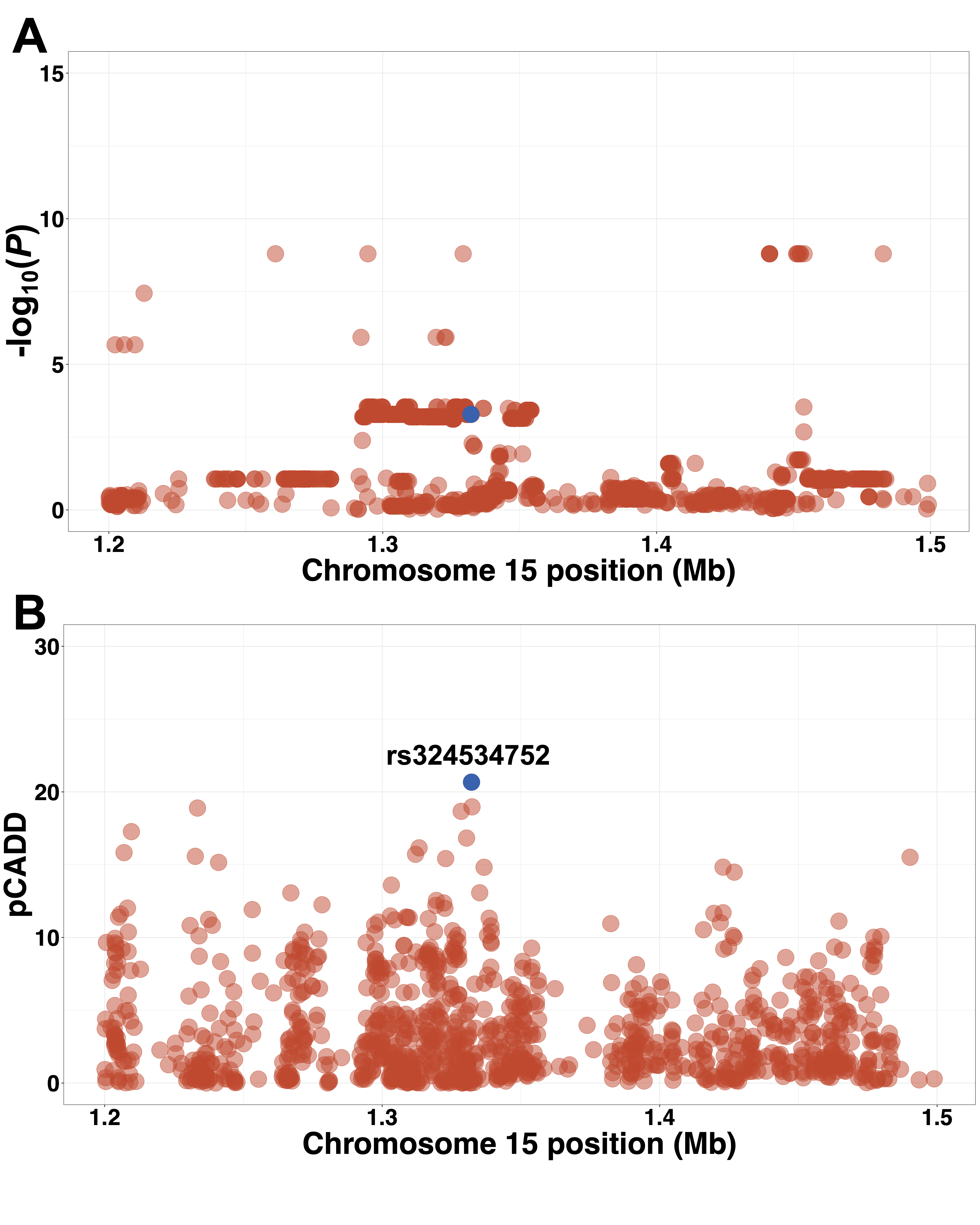

Supplement: Supplementary file 8 — Additional file 8: Fig. S8. Regional association plots around rs324534752. (A) GWAS result around rs324534752. (B) pCADD values around rs324534752. [file 40104_2022_751_MOESM8_ESM.png]

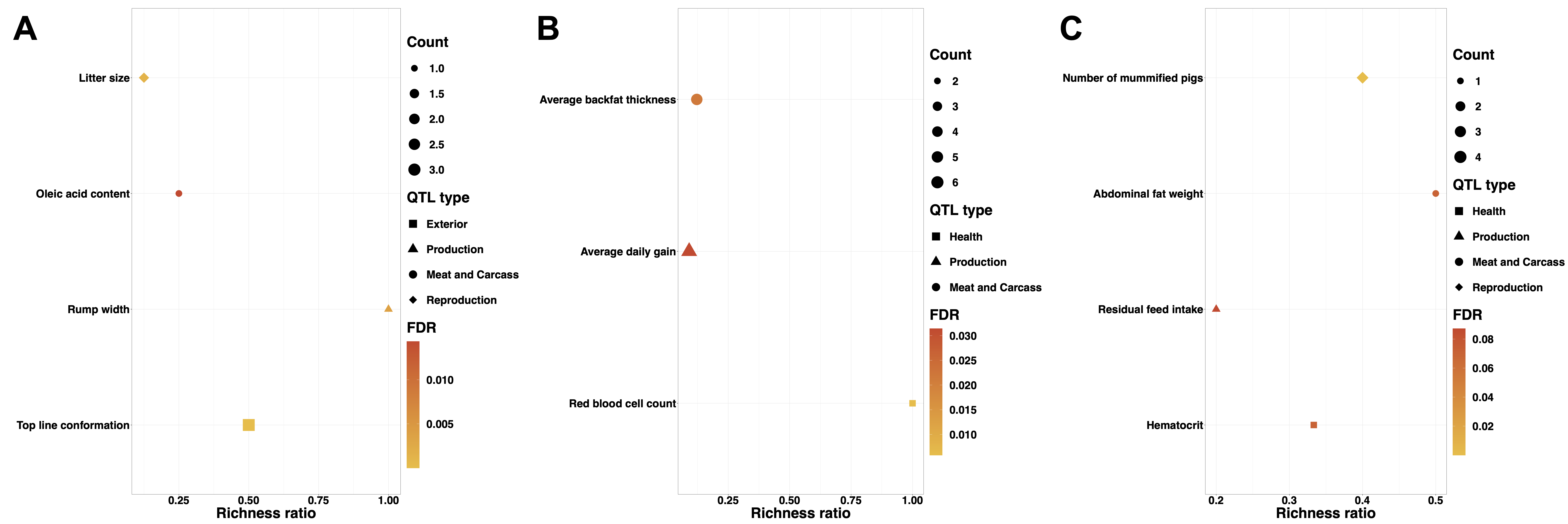

Supplement: Supplementary file 9 — Additional file 9: Fig. S9. QTL enrichment results of the potential selection regions around rs346331089 (A), rs322980623 (B), and rs324534752 (C). [file 40104_2022_751_MOESM9_ESM.png]
